# Supplementary material for: Anti-ApoA-1 IgGs in Familial Hypercholesterolemia Display Paradoxical Associations with Lipid Profile and Promote Foam Cell Formation
Source: J Clin Med. 2019 Nov 21;8(12):2035. doi: 10.3390/jcm8122035 (PMC6947407; doi:10.3390/jcm8122035)
Supplement: Supplementary file 1 [file jcm-08-02035-s001.pdf]

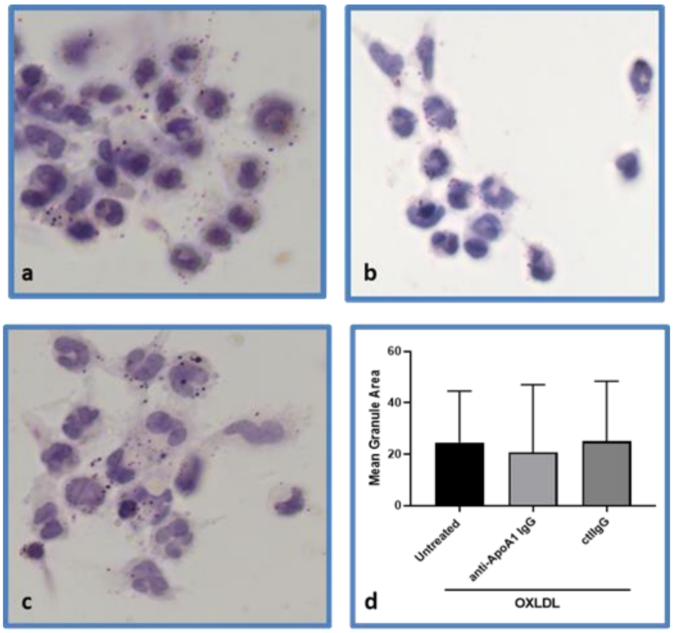

Supplemental Figure 1. Anti-ApoA-1 IgGs don't promote oxLDL uptake. HMDM were treated for 24 hours with oxLDL at 20µg/mL (panel a) in presence of anti-ApoA-1 IgG (panel b) or control IgG (panel c). Cells were stained with Oil Red-O to highlight the lipid uptake. Oil Red-O staining quantification as the mean granule area per cell. Oil Red-O was quantified as indicated in the method section. In panel d the results are expressed in arbitrary units as mean±SD of four independent experiments (n = 4).

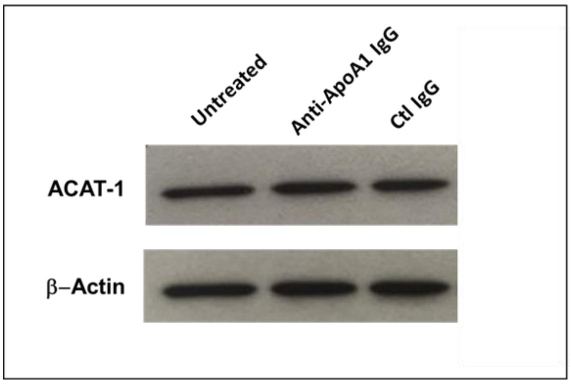

Supplemental Figure2. ACAT-1 expression is not modulated by anti-ApoA-1 IgG. One of three representative western blot is shown. ACAT-1 expression is unchanged after anti-ApoA1 IgG stimulation compared to the untreated or ctl IgG condition.

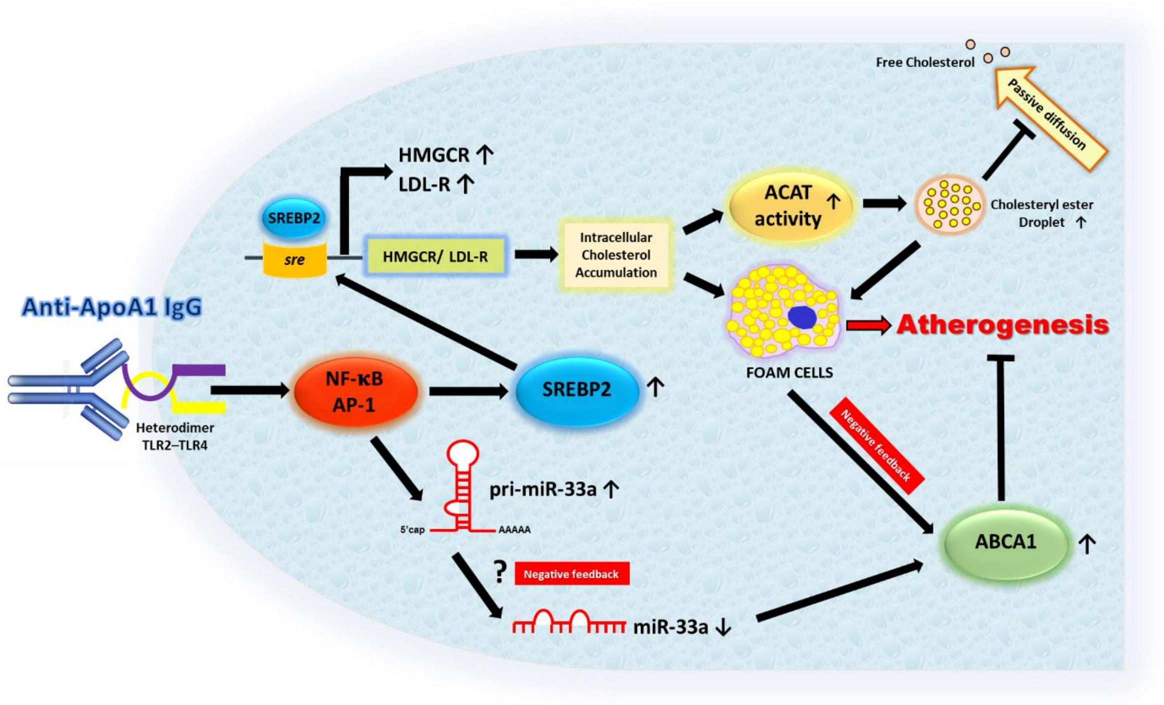

Supplemental Figure 3. Summary of the anti-ApoA-1 IgG-mediated foam cell formation. Anti-ApoA-1 antibodies bind the heterodimer TLR2/TLR4 activating NF-kB and AP-1 pathway enhancing the transcription factor SREBP2 specific for the HMGCR and LDL-R transcriptional activation leading to an increase of intracellular cholesterol level increasing the ACAT activity which shifts the membrane free-cholesterol within cytosolic esterified lipid droplets, decreasing the free-cholesterol gradient and the associated passive diffusion cholesterol efflux culminating with the pro-atherogenic foam cell formation. In response to this lipid accumulation, a compensatory negative feedback loop is activated involving an increased ABCA1 expression, insufficient to counteract the anti-ApoA-1 IgG pro-atherogenic effects. Whether the anti-ApoA-1 IgG-associated downregulation of miR-33a is a direct or indirect effect part of such compensatory mechanism remains unknown.
